# Stable Isotope Ratio Analysis for Authentication of Natural Antioxidant Cannabidiol (CBD) from *Cannabis sativa*

**DOI:** 10.3390/antiox12071421

**Published:** 2023-07-14

**Authors:** Matteo Perini, Alessio Gaggiotti, Silvia Pianezze, Luca Ziller, Roberto Larcher

**Affiliations:** 1 Fondazione Edmund Mach, Via E. Mach 1, 38098 San Michele all’Adige, Italy; matteo.perini@fmach.it (M.P.); luca.ziller@fmach.it (L.Z.); roberto.larcher@fmach.it (R.L.); 2 Farmech, Piazza Duomo 20, 20122 Milano, Italy; consorziosantandrea@gmail.com

**Keywords:** cannabidiol, *Cannabis sativa*, stable isotope ratio analysis, biosynthetic, authentication

## Abstract

Cannabidiol (CBD) is a non-psychoactive cannabinoid of *Cannabis sativa* that exhibits several beneficial pharmacological effects, including anti-inflammatory and antioxidant properties. The molecule can be obtained via extraction from the plant or through a biosynthetic route. The two products have both advantages and disadvantages, thus necessitating the development of methods capable of distinguishing between the two products. In this study, for the first time, the analysis of the stable isotope ratios of oxygen and hydrogen demonstrated high efficiency in the discrimination of CBD of a totally natural origin from that obtained through chemical synthesis. Considering a probability level of 95%, it was possible to identify threshold values for *δ*^2^H and *δ*^18^O of the totally natural CBD of −215‰ and +23.4‰, respectively. Higher values may indicate a non-entirely natural origin of CBD (i.e., a biosynthetic molecule).

## 1. Introduction

Cannabidiol (CBD) is one of the over one hundred and forty identified phytocannabinoids in the *Cannabis sativa* plant, which was isolated, for the first time, in 1940 by Adams et al. through its ester *bis*-3,5-dinitrobenzoate from marijuana red oil [1,2]. *Cannabis sativa* still represents the main, if not the only, natural source from which the molecule can be extracted. Indeed, Appendino et al. demonstrated that the reported isolation from hops (*Humulus lupulus L. Cannabaceae* and *Humulus Kriya* (sic)) was fraudulent, and that the one from *Trema orientalis* and stevia was dubious, while the presence of trace amounts of CBD in flax (*Linum usitatissimum L. Linaceae)* requires additional confirmation [3]. On the other hand, another important cannabinoid, cannabigerol (CBG), along with its acidic form (cannabigerolic acid), have been detected in South African *Helichrysum umbraculigerum Less. Asteraceae* [4,5]. 

As a cannabinoid derived from *Cannabis sativa*, CBD is the subject of controversy regarding its legal use in light of the different interpretations and applications of international drug control treaties. As demonstrated in 1970 by Mechoulam et al. [6], CBD does not have the same degree of psycho-activity as tetrahydrocannabinol (THC), which is the main psychotropic agent in *Cannabis sativa*. The progressive and partial liberalization of the plant, which started at the beginning of the 21st century, and the WHO’s listing of CBD as a ‘not controlled’ substance in 2017 [7], allowed for the launching of a great deal of research on the potential pharmacological uses of CBD and, in some countries, the marketing of products containing it. In recent years, researchers conducted human laboratory studies and clinical trials (for example, randomized controlled trials and single-arm, open-label studies) evaluating the administration of CBD as a therapy for various medical conditions, including epilepsy [8], anxiety [9], pain/inflammation [10], schizophrenia [11], various substance use disorders [12], and others [13]. While there is strong evidence supporting the usefulness of CBD for the treatment of epilepsy [14], for other pathologies, the effectiveness of CBD often remains conflicting and/or there is a lack of well-powered, randomized, placebo-controlled trials from which to draw firm conclusions. 

Moreover, several studies recently demonstrated the antioxidant properties of cannabidiol [15]. CBD has been shown to affect the redox balance by changing the levels and activity of both oxidants and antioxidants. CBD disrupts free radical chain reactions by either capturing free radicals or transforming them into less-active forms; it also reduces oxidative conditions by preventing the formation of superoxide radicals, and it reduces the production of reactive oxygen species (ROS). From a chemical point of view, the hydroxyl groups of the phenolic ring of the molecule seem to be mainly responsible for the antioxidant activity of CBD.

In addition to natural CBD obtained from *Cannabis sativa* plants, the synthetic CBD produced using chemical (i.e., via synthetic pathway) or biological processes conducted in a laboratory, such as the use of yeast (i.e., via bio-synthetic pathway), is now widely available on the market. To date, there is no regulation—in the pharmaceutical field—forbidding the use of synthetic products instead of natural ones; therefore, it is possible to find products containing both types of CBD. There is an ongoing discussion in the *Cannabis*-industry about the pros and cons of synthetic and natural CBD. The main problem lies in the differences between the two forms in terms of efficacy, safety, consumer preferences, cost-effectiveness, and legality. Some argue that the synthesis of CBD in a laboratory requires less-expensive resource inputs than field-grown hemp, but on the other hand, the price of natural CBD on the market is subject to significant fluctuations, which can make it even cheaper than the synthetic form [16]. Some studies seem to demonstrate that the synthetic route allows for the acquisition of a pure product without the problems linked to the extraction process from *Cannabis sativa* plants, such as the presence in the extract of traces of THC [17]. On the other hand, Citti et al. recently showed that the presence of carbon dioxide and water from the air could be the key to create the acidic environment responsible for the cyclization of CBD into THC, which is detected as an impurity even in batches of the synthetic product [18]. Some studies have recently reported that the presence of other compounds in the *Cannabis*-derived extract, which are absent in the synthetic molecule, can support the benefits of CBD though a phenomenon called the “entourage effect” by bolstering, for instance, the calming or antiepileptic effects of CBD [19,20]. Regarding consumers’ preferences, the natural product represents the favored option, and this means that *Cannabis*-derived products will dominate the market for the foreseeable future [21]. 

In this context, and mainly to ensure that consumers (both doctors for their clinical trials and patients using the cannabinoid) are aware of the type of product they are using, it is necessary to develop analytical methods that allow one to unequivocally distinguish natural CBD from that obtained through biosynthetic or synthetic means. To date, analytical methods that allow for the different origins of CBD to be differentiated have not been reported in the literature. The stable isotope analysis of bio-elements has already been successfully tested as a technique for distinguishing different natural molecules from their synthetic and/or biosynthetic counterparts. These molecules include, e.g., monacolin K from fermented red rice [22], theanine [23], curcuminoids [24], and vanillin [25]. The ^13^C/^12^C isotope ratios of carbon (expressed as *δ*^13^C), the ^18^O/^16^O ratios of oxygen (*δ*^18^O), and the ^2^H/^1^H ratios of deuterium (*δ*^2^H) proved to be effective in the discrimination between the active principal compound of interest obtained from natural sources and that obtained from fossil sources used in the industrial synthesis. For the first time, in this study, natural CBD and the synthetically produced form were characterized according to their different stable isotopic ratios. 

## 2. Material and Methods

### 2.1. Sampling

Twenty-five samples of *Cannabis sativa* inflorescences were supplied by the certified mass producer Farmech Srl (Milano, Italy) to three selected labs in charge of extracting and purifying cannabidiol, whose researchers followed the extraction protocol described in Section 2.2 and measured the CBD concentration in the final product. Six samples of ≥98.0% synthetic cannabidiol were purchased on the market. The limited number of global producers from whom this product can be purchased, all of whom are located in the United States and China, justify this sample size. At the Edmund Mach Foundation (FEM, San Michele all’Adige, Italy), only isotopic analyses were performed on the cannabidiol samples, supplied by the producers, with a declared purity of not less than 90%. Furthermore, six blends of cannabidiol supplemented with different concentrations of synthetic CBD were prepared in an FEM analysis laboratory. The samples were freeze-dried and ground to a powder.

### 2.2. Protocol for the Natural Production of Cannabidiol

In brief, the samples of raw material (*Cannabis sativa*) are subjected to an initial analytical examination to determine the content of cannabinoids, metals, pesticides, and other toxicants. The plant material is then ground through a 2 mm mesh and then stored in a sealed bag at room temperature prior to its use. Since the composition of the plant material can change during the storage, it must be characterized before each experiment. The stages of the process include a cold extraction with ethanol, winterization, extraction with hexane, decarboxylation, and crystallization. 

#### 2.2.1. Cold Extraction with Ethanol

Due to its efficiency, the “cold process” is the preferred large-scale method for the ethanol-based extraction of CBD from *Cannabis sativa*. The US patent US9987567B1 partially discloses some parts of the extraction protocol. *Cannabis sativa* flowers are ground and dipped in ethanol at a temperature between −40 °C and −50 °C for fifteen minutes under pressure. The degree of solvency is determined, and then winterization is carried out.

#### 2.2.2. Winterization

Winterizing is a process that removes unwanted elements extracted from hemp, such as fats, waxes, and lipids, leaving clean and consumable CBD oil behind. The extract is dissolved in ethanol using a ratio of 10:1 mL:g (ethanol/extract). The solution is stirred using a laboratory spatula or a magnetic stirrer/heater with a stir bar. The waxes, lipids, and fats are thus dissolved in the solution. The solvent is cooled to decrease the solubility and to ensure the precipitation of the waxes. The solution is chilled, using a refrigerator/freezer, for at least 24 h. The solution is then filtered under vacuum with a Buchner funnel and filter paper. Using a hotplate, the ethanol is boiled until the solution has reached a thicker viscosity. If a vacuum oven is used, the boiling point of the ethanol is reduced to 12.8 °C. This process purifies the *Cannabis sativa* solution, resulting in the maximum oil yield with the lowest number of impurities.

#### 2.2.3. Extraction with Hexane—Decarboxylation 

The sample is extracted with hexane and then dried. The dry extract is placed in an incubator oven and incubated at 150 °C for 60 min to induce the decarboxylation of the acidic cannabinoids into their neutral forms. The decarboxylation process consists in removing the carboxylic acid and CO_2_ from the cannabinoids present in the *Cannabis* extract.

#### 2.2.4. Crystallization

The mixture is heated while being constantly stirred. The temperature is then lowered, and the stirring speed is decelerated. Once the mixture has cooled and nucleation (the initial stages of crystallization) has begun, the stirring speed is increased dramatically, causing the crystals to separate from the solution. Next, the crystals are rinsed with hexane, or another chemical solvent, to remove any remaining unwanted impurities. The crystals are then dried and tested with regard to their purity, which must be greater than 90%.

### 2.3. Stable Isotope Analysis

The CBD crystal samples were received in this form from the FEM laboratory and analyzed therein after being ground and freeze-dried. The ^13^C/^12^C ratio was measured using an isotope mass spectrometer (IsoPrime, Isoprime Limited, c) after total combustion in an elemental analyzer (VARIO CUBE, Isoprime Limited, Frankfurt, Germany). The ^2^H/^1^H and ^18^O/^16^O ratios were measured using an IRMS (Finnigan DELTA XP, Thermo Scientific, Waltham, MA, USA) coupled with a pyroliser (Finningan DELTA TC/EA, high-temperature conversion elemental analyzer, Thermo Scientific, Waltham, MA, USA). When analyzing the samples, the amounts introduced in the mentioned instruments were 0.5 and 0.2 mg, respectively.

In the EA, the samples are brought to a temperature of 1020 °C in an atmosphere rich in O_2_ for a few seconds to enable complete combustion (“flash burning”). The gases produced are transported, via a constant flow of carrier gas (helium), into a further oxidation column made up of catalysts (CuO, Cr_2_O_3_, and Co_3_O_4_-Ag), allowing for the abatement of CO (conversion into CO_2_). The resulting gaseous mixture is composed of CO_2_, N_2_, N_x_O, and H_2_O. The excess oxygen is removed by passing these gases through a second quartz column, filled with metallic Cu, at 680 °C in order to reduce the N_x_O to N_2_. The H_2_O molecules are eliminated through a Mg(ClO_4_)_2_ filter.

The pyrolysis process on which the TC/EA-IRMS measurement is based is rapid and quantitative and occurs when a reducing environment is created at high temperatures (typically 1400 °C). The reactors used in this regard consist of glassy carbon tubes in order to prevent the samples or reactive gases from coming into contact with the oxygen-containing surface (e.g., Al_2_O_3_).

After the water has been removed, the gases N_2_, CO_2_ and SO_2_ (as for the EA-IRMS) or CO and H_2_ (as for the TC/EA-IRMS) are separated using a gas chromatography (GC) column, and the various isotope ratios are analyzed in sequence. The column exploits a particular type of stationary phase called “mixed”, consisting of porous polymers, that separates the different gases through a process of adsorption and partition. Afterward, through a universal flow-through interface, they reach the IRMS.

According to the IUPAC protocol [26], ^13^C/^12^C, ^2^H^/1^H, and ^18^O/^16^O values are expressed in the delta scale (*δ*‰) against the international V-PDB (Vienna-Pee Dee Belemnite) standard according to Equation (1):

(1) $${\delta_{ref}(}^{i}E{/}^{j}E,sample)=\left[\frac{R{(}^{i}E{/}^{j}E,sample)}{R{(}^{i}E{/}^{j}E,ref)}\right]-1$$

where *ref* is the international measurement standard, *sample* is the analyzed sample, and *^i^E/^j^E* is the isotope ratio between heavier and lighter isotopes. The delta values were multiplied by 1000 and expressed in units “per mil” (‰).

The isotopic values were calculated against working in-house standards (caseins), which were themselves calibrated against international reference materials: fuel oil NBS-22 with *δ*^13^C = −30.03‰, sucrose IAEA-CH-6 with *δ*^13^C = −10.45‰ (IAEA-International Atomic Energy Agency, Vienna, Austria), and L-glutamic acid USGS 40 with *δ*^13^C = −26.39‰ (U.S. Geological Survey, Reston, VA, USA) for ^13^C/^12^C. 

Before carrying out the analysis of *δ*^2^H, the sample was left to equilibrate with the laboratory air for two days; subsequently, it was placed inside a vacuum dryer with P_2_O_5_ for 48 h and, finally, placed in a zero-blank autosampler to comply with the principle of identical treatment. USGS 84 (Olive oil from Sicily, Italy, *δ*^2^H = −140.4 ± 3.1‰, *δ*^18^O = +26.36 ± 0.50‰) and USGS 86 (Tropical Vietnamese peanut oil, *δ*^2^H = −207.4 ± 4.5‰, *δ*^18^O = +18.76 ± 1.03‰) acquired from the U.S. Geological Survey were used to obtain the ^2^H/^1^H and ^18^O/^16^O values. Geological surveys were used to obtain ^2^H/^1^H values through the creation of a linear equation and by adopting a comparative equilibration procedure [27]. We used these two olive oil standards because of the absence of any international organic reference material with a matrix similar to that of our samples (cannabidiols). The uncertainty of the method (calculated as two standard deviations when analyzing the same sample at least ten times under reproducible conditions) was 0.3‰ for *δ*^13^C, 0.5‰ for *δ*^18^O, and 4.0‰ for *δ*^2^H.

### 2.4. Statistical Analysis

The data were statistically evaluated using XLSTAT (XLSTAT, 2017, Paris, France). The existence of differences was verified through regression analysis at a confidence level of 95%. One-way ANOVA was performed to determine the significant spatial difference of variables. Tukey’s Honest Significant Difference (HSD) test for unequal sample sizes was used to evaluate significant differences due to geographical origin. A test probability value (*p* < 0.05) was used to indicate significance levels.

## 3. Results and Discussion

### 3.1. Stable Isotope Variability of Natural and Synthetic Cannabidiol

Although CBD is mainly obtained through organic solvent extraction from plant sources [28], products of non-completely natural (biosynthetic) origin are now available on the market. Since Adams et al. identified the molecular structure of CBD in the 1940s [1], various synthetic strategies for obtaining CBD have been proposed [29,30], including Mechoulam and Gaoni’s first synthesis of racemic (±)-CBD from citral [31]. Today, the synthesis strategies applied to produce racemic or enantiopure CBD allow us to classify CBD analogues into three groups: C4’, resorcinol, and limonene analogues [32]. Regardless of the biosynthetic route adopted, the only difference is the monoterpenic precursor of a natural origin, which then condenses with olivetol (synthetic). The most used compounds derive from carene and limonene (menthadienol, isopulegol, and verbenol). Therefore, biosynthetic CBD does not derive from fossil sources exclusively; rather, part of the molecule is derived from natural sources. Considering the CBD formula (C_21_H_30_O_2_) and that of its fossil origin precursor olivetol (C_11_H_16_O_2_), it is worth noting that the latter supplies to the former about 50% of its carbons and hydrogens (while the remaining 50% has a natural origin) and 100% of its oxygens. 

The *δ*^13^C of the fully natural CBD ranged between −31.3‰ and −28.4‰, with an average value of −30.4 ± 0.8‰ (Table 1). These properties align with the botanical origin of the matrix. Indeed, *Cannabis sativa* belongs to the group of plants with a C3 photosynthetic cycle whose *δ*^13^C ranges from −29 to −25‰ [33]. Regarding the specific plant used, Denton et al. reported values between −29‰ and −25‰ for *Cannabis sativa* from Queensland, Australia, Papua New Guinea, and Thailand [34], while in the study by Shibuya et al., samples of *Cannabis sativa* from Brazilian dry regions ranged from −28 to −25%, and those from humid regions varied from −32 to −25% [35]. For Hurley et al., the *δ*^13^C of the plants collected in various areas of the USA varied from −35‰ to −29‰ [36], while for Calvi et al. the *δ*^13^C for Italian *Cannabis sativa* ranged between −30.9 to −23.6‰ [37]. 

The CBD obtained through the biosynthetic route had a range of variability of *δ*^13^C between −31.8‰ and −30.1‰ (average −31.3 ± 0.6‰, Table 1), which was not statistically different from the natural one (*p* > 0.05). As reported by Focella et al., the olivetol used to obtain biosynthetic CBD is usually synthetized on a large scale through the reaction of an a,b-unsaturated ketone with dimethyl malonate enolate [38]. As already observed in other totally synthetic molecules isolated from fossils (such as synthetic vanillin), the isotopic value *δ*^13^C of these precursors is around −30‰ [25]. In the study by Yeh et al., products from fossils showed a typical variability range between −32.5‰ and −23.3‰ [39]. Falling in the same range of variability, the analyzed synthetic and biosynthetic molecules cannot be discriminated through carbon isotope analysis.

The isotopic ratios of hydrogen and, to an even greater extent, oxygen, in totally natural CBD are instead significantly different from those observed in CBD obtained through biosynthesis. In the natural product, the *δ*^2^H varies from −274‰ to −227‰, while in the biosynthetic product, this value ranges between −206‰ and −135‰ (Table 1). The *δ*^2^H measured in the natural CBD is strictly correlated with that of the original plant *Cannabis sativa*, whose isotopic composition reflects that of the ground water absorbed through the roots. The isotopic signature of rainfall influences the hydrogen isotopic composition of ground water, as reported by Gat et al. [40]. Hurley et al. reported a range of *δ*^2^H variability of American *Cannabis sativa* from −160‰ to −129‰ [36], while in Italian samples the values range between −200‰ and −68‰ [37]. The gap between the higher *δ*^2^H value of *Cannabis sativa* and the lower value of natural CBD can be explained by the fact that the different compounds have characteristic isotopic compositions due to the different metabolic pathways involved in their synthesis. The same behavior has already been observed in other molecules of a vegetable origin, such as Monacolin K. While the rice from which the molecule is derived varies from −71‰ to −27‰ [41], monacolin K has an average value of −259 ± 6‰ [22]. 

Normally, it is possible to correlate the *δ*^2^H value of the molecule under study (i.e., natural CBD) with that of the groundwater absorbed by the plants (in this case, *Cannabis sativa*) in the location where the plants grew. Having no information about the exact origin of the plants used for production, it was not possible to evaluate the correlation between CBD and its geographical origin. Instead, it was possible to note how the source, despite representing 50% of the hydrogens from the fossil precursor in the biosynthetic CBD molecule, significantly increased the *δ*^2^H (an average of −171 ± 25‰, which is higher than that of −244 ± 15‰ of the natural form). The biosynthetic pathways adopted in the industrial production of the precursor olivetol resulted in a product characterized by a specific hydrogen isotopic composition. A similar behavior has already been observed for other synthetic molecules such as vanillin [25]. In the present study, the effect is less evident since the biosynthetic molecule is just partially obtained from fossil precursors. Therefore, the *δ*^2^H value is influenced both by the δ^2^H of synthetic olivetol (probably with high δ^2^H) and by the δ^2^H of natural precursors (which are more likely to have lower values). Considering a probability level of 95%, it was possible to identify a threshold value for *δ*^2^H of the totally natural CBD of −215‰. Higher values may indicate a non-totally natural origin of the CBD.

Regarding the *δ*^18^O, the mean value of the natural CBD (+20.1 ± 1.7‰) and the biosynthetic one (+26.9 ± 0.6‰) resulted significantly different (*p* < 0.01) (Table 1). In the latter, both oxygens of the molecule derive from the synthetic precursor olivetol. Therefore, the *δ*^18^O is less influenced by the natural component present in the biosynthetic sample than the other investigated isotopic ratios (carbon and hydrogen). The *δ*^18^O can, therefore, be considered the most powerful parameter in the discrimination between the two types of products. For *δ*^18^O, it was possible to identify a threshold value (95% probability) of natural CBD equal to +23.4‰. Higher values may indicate that the CBD is not of an entirely natural origin. 

### 3.2. Natural Cannabidiol Spiked with Different Concentrations of Synthetic CBD

**Figure 1:**
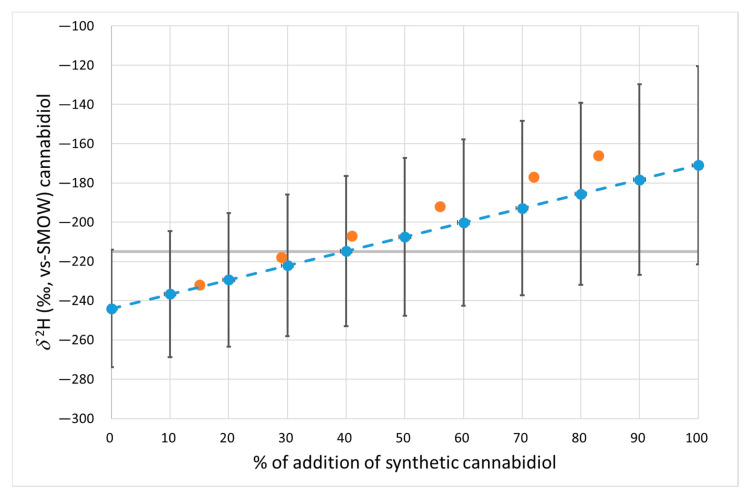
Variations in the *δ*^2^H values of natural CBD with the addition of biosynthetic CBD. The grey line defines the 95% threshold limit for authentic natural CBD. Blue dots: mean value. Bars: 95% confidence limit. Orange dots: measured values of the prepared mixtures.

**Figure 2:**
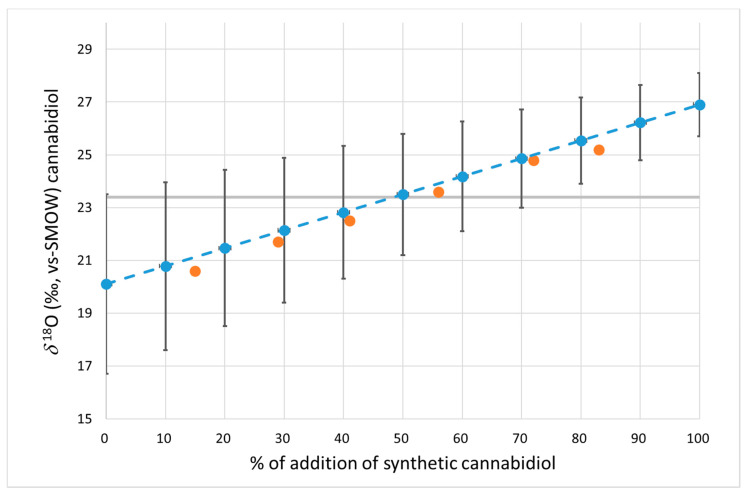
Variations in the *δ*^18^O values of natural CBD with the addition of biosynthetic CBD. The grey line defines the 95% threshold limit for authentic natural CBD. Blue dots: mean value. Bars: 95% confidence limit. Orange dots: measured values of the prepared mixtures.

To quantify the percentage of cannabidiol of synthetic origin added to a sample of natural cannabidiol, graphs that correlate the isotopic value *δ*^2^H (Figure 1) and *δ*^18^O (Figure 2) with the percentage of adulterant added (from 0% to 100%) were created. The graphs indicate the expected average values obtained from the mixture of natural and synthetic CBD in the different percentages reported. As reported in Table 1, natural and synthetic CBD do not have a single stable isotope value for *δ*^2^H and *δ*^18^O but rather a range of variability. For this reason, variability bars calculated as standard deviations were indicated for each mean value (multiplied by t-student) of the two groups. The mean values of the blend were calculated as the sum of the mean values for the two groups multiplied by the contribution percentage to the blend, while the standard deviation was the sum of the standard deviation of the two groups multiplied by the percentage of contribution, according to the law of propagation of error in the case of the sum of two or more variables. The validity of the graph was confirmed through an analysis of six blends obtained by adulterating a sample of natural cannabidiol (*δ*^2^H = −246‰ and *δ*^18^O = +19.2‰) with increasing amounts (from 15% to 83%) of synthetic CBD (*δ*^2^H = −150‰ and *δ*^18^O = +26.5‰). The values of these six samples were all within the variability limits described by the correlation lines (for *δ*^2^H and *δ*^18^O, respectively) and are shown as orange circles in Figure 1 and Figure 2. 

The 95% limit values (see Table 1) are also indicated in the graphs (gray dotted lines in Figure 1 and Figure 2). As shown in the graphs, the isotopic ratios *δ*^2^H and *δ*^18^O allowed for the identification of the synthetic adulterant with percentages of additions higher than 40% and 50% on average, respectively. As it can be seen from the graph, this percentage was calculated in relation to the hypothesis of a mixture between a natural and a synthetic sample with values equal to the average values reported for the two groups (−171‰ vs −244‰ for *δ*^2^H and +20.1‰ vs +26.9‰ for *δ*^18^O). Different values of the two starting CBDs (natural and synthetic) can lead to an increase or decrease in this percentage. When evaluating the data, it will also always be necessary to account for analytical uncertainty (which was significantly greater for *δ*^2^H than for *δ*^18^O).

Regarding the isotopic value *δ*^2^H, the high spike identification threshold observed is mainly due to the fact that the synthetic molecule partially originates from a natural source. In fact, biosynthetic CBD derives from the combination of fossil origin (e.g., olivetol) component and a natural origin one. This evidence does not allow for the identification of significant differences in terms of δ^2^H, as it has already been seen in other studies on organic molecules (e.g., vanillin or curcuminoids complex) [24,25].

Regarding the *δ*^18^O, the range of variability of the natural product was significantly different from that of the synthetic form, thus allowing for a clear discrimination between the two types of CBD (see Table 1 and Section 3.1). However, the difference between the average values of the two types of CBD (about 6‰) is not enough to provide the required ability with which to identify the cuts, which are detectable, on average, only with the addition of synthetic CBD higher than 50%.

Considering the absence, to date, of analytical methods capable of discriminating between the two types of cannabinoids, we believe that most samples available on the market may be counterfeits (i.e., synthetic products passed off as natural products) and that they can be easily identified through the proposed analytical method. The expected diffusion of increasingly sophisticated adulterations, such as mixes between natural and synthetic products, which are difficult to be detected through the stable isotope ratio analysis of bulk, will certainly require the development of new analytical approaches. For instance, the SNIF-NMR compound specific isotope analysis could allow for the analysis of the different isotopic ratios in the individual parts of the molecule under study. 

## 4. Conclusions

Cannabidiol is certainly one of the most interesting antioxidants and anti-inflammatories on the market today. The exponential increase in its demand and the liberalization introduced in many states since the early 2000s have flooded the market with low-priced products of uncertain origin. In particular, CBD of biosynthetic origin has shown various critical aspects; therefore, it is mandatory to develop increasingly optimized tools and technologies capable of discriminating the synthetic and the natural product. In this study, authentic samples of natural origin and biosynthetic samples were analyzed. The stable isotope ratios of hydrogen and, in particular, of oxygen demonstrated an excellent discriminating ability, with 95% cutoff values for the all-natural CBD of −215‰ and +23.4‰, respectively. The identification of adulterated products obtained by mixing natural and synthetic CBD is more difficult due to the small difference between the mean value of the hydrogen-to-oxygen isotope ratio of natural versus synthetic CBD. Considering the average values of the two groups, it is possible to identify additions higher than 40% with the use of the isotopic parameter *δ*^2^H and higher than 50% using *δ*^18^O. Therefore, isotope ratio analysis lends itself primarily to pure product testing, and it can be an effective answer as it is a relatively rapid technique that is performed routinely by several laboratories around the world. 

## Figures and Tables

**Table 1:** Type of samples, the laboratory (a, b and c) that performed the extraction and purification, and the experimental values (mean, SD, min and max, and low and high threshold values at 95%) of the *δ*^13^C, *δ*^2^H, and *δ*^18^O stable isotopic parameters of natural and biosynthetic CBD.

Type of Sample	N. Sample	Extraction Laboratory	*δ*^13^C (‰. Vs. V-SMOW)	*δ*^2^H (‰. Vs. V-SMOW)	*δ*^18^O (‰. Vs. V-SMOW)
Naturally extracted CBD	1	b	−30.3	−274	18.0
	2	c	−30.7	−272	19.2
	3	b	−29.6	−268	14.9
	4	b	−30.1	−260	18.7
	5	c	−30.2	−260	18.8
	6	a	−30.7	−259	19.8
	7	c	−29.8	−258	18.0
	8	b	−30.7	−251	20.9
	9	b	−28.4	−250	20.7
	10	a	−31.2	−246	19.2
	11	c	−29.9	−243	18.5
	12	a	−31.1	−239	19.8
	13	a	−29.0	−238	19.2
	14	b	−28.6	−231	22.1
	15	b	−31.0	−236	22.0
	16	a	−30.9	−236	20.7
	17	a	−31.0	−236	20.9
	18	a	−30.9	−235	21.2
	19	c	−31.0	−235	20.7
	20	a	−31.0	−234	21.2
	21	c	−31.1	−233	20.7
	22	c	−31.3	−231	22.0
	23	b	−30.0	−230	21.6
	24	c	−31.3	−227	22.0
	25	b	−30.4	−227	20.9
	mean		−30.4	−244	20.1
	SD		0.8	15	1.7
	min		−31.3	−274	14.9
	max		−28.4	−227	22.1
	limit 95% min		−32.1	−273	16.7
	limit 95% max		−28.8	−215	23.4
Biosynthetic CBD	26		−31.8	−206	26.3
	27		−31.7	−183	26.9
	28		−31.3	−180	27.3
	29		−30.1	−135	27.8
	30		−31.2	−172	26.6
	31		−31.5	−150	26.5
	mean		−31.3	−171	26.9
	SD		0.6	25	0.6
	min		−31.8	−206	26.3
	max		−30.1	−135	27.8
	limit 95% min		−32.5	−222	25.8
	limit 95% max		−30.0	−121	28.0

## Data Availability

Data is contained within the article.
